# Delivering on the promise of early detection with liquid biopsies

**DOI:** 10.1038/s41416-021-01646-w

**Published:** 2022-01-10

**Authors:** David Crosby

**Affiliations:** Prevention and Early Detection Research, Cancer Research UK, London, UK

**Keywords:** Cancer, Cancer screening, Cancer screening

## Abstract

Liquid biopsy approaches are relatively well developed for cancer therapy monitoring and disease relapse, but they also have incredible potential in the cancer early detection and screening field. There are, however, several challenges to overcome before this potential can be met. Research in this area needs to be cohesive and, as a driver of research, Cancer Research UK is in an ideal position to enable this.

## Main

We know that cancer is easier to treat, and causes patients to suffer less and live longer when it is detected at an earlier stage [1]. That is why Cancer Research UK is trying to reach a position whereby no cancer is detected late.

The past few decades have seen the promise of non-invasive techniques to examine tumour biology begin to deliver [2]. As such, liquid biopsy approaches will be an important part of the mix of techniques, which will help us reach our early detection goal allowing patients to get their cancer diagnosis as early as possible.

Despite advances, invasive tumour biopsy remains the primary method of diagnosis. However, the limitations of utilising this approach for the early detection of solid tumours are well known. Considerable difficulties in accessing certain tumours, changes in tumour genetic composition arising from malignant neoplasms [3] and intra-tumour heterogeneity can be problematic for conventional tumour biopsy [4].

The minimally invasive nature of liquid biopsy offers the potential of relatively easy repeat sampling over time. It is clear then that the application of liquid biopsy-based early detection is a vital direction of travel for the research community.

## How can we enable this?

As a funder and driver of research in this important area, our aim is to enable the research community to make significant, and cohesive, progress. Our recent *Early Detection and Diagnosis of Cancer Roadmap* points out how the early detection and diagnosis ecosystem can meet the pressing need to increase our ability to accurately detect and diagnose cancer at an early stage to transform health outcomes (https://www.cancerresearchuk.org/sites/default/files/early_detection_diagnosis_of_cancer_roadmap.pdf). It encompasses a wide range of approaches, including a call to arms for those developing new technologies and methods to drive progress in this area.

Because Cancer Research UK was an early supporter of work in this field, we see the importance of funding liquid biopsy research to yield important results. In 2013, CRUK researchers Carlos Caldas and Nitzan Rosenfeld, based at our Cambridge Institute, were collaborators on a pivotal proof-of-concept study showing circulating tumour DNA (ctDNA) has a higher sensitivity to that of circulating tumour cells (CTCs) as a biomarker of metastatic breast cancer [5]. The study was focussed on the potential of this approach to monitor therapy in patients and found that ctDNA levels showed a greater dynamic range and greater correlation with changes in tumour burden than CTC.

In the same year, Rosenfield and Caldas went on to co-author another study—one of the first to show that genomic alterations in solid cancers can be characterised by sequencing ctDNA [6]. The work showed that sequencing of cancer exomes in serial plasma samples can be used to track the genomic evolution of metastatic cancers in response to therapy. It also established proof of principle that exome-wide analysis of ctDNA could complement invasive biopsy approaches to identify mutations associated with acquired drug resistance in advanced cancers.

One of the remaining challenges to this, however, is that the sensitivity of ctDNA analysis is limited by the amount of tumour DNA in the blood. When amounts of ctDNA in plasma are low, as they often are in early-stage cancers, analysis can be unreliable because the signal is below the limit of detection [7]. Recent work by CRUK researchers suggests that a patient’s individual tumour sequence data can be used to overcome this [8]. In the study, they developed a method for sensitive patient-specific monitoring of ctDNA that utilises the properties of patient-specific sequencing data. They describe the INtegration of VAriant Reads (INVAR) approach, which uses custom error suppression and signal-enrichment methods to increase the sensitive monitoring and identification of residual disease. Whilst these studies were not focussed specifically on early detection, they can be seen as a stepping stone to that end.

## Multiple-cancer potential

The development of multi-cancer early detection (MCED) tests has generated a lot of recent attention. And it is clear to see why. A single test for simultaneous detection of multiple cancers is an exciting prospect for early detection and screening and there is evidence to suggest the approach will have utility. Modelling work suggests that 26% of cancer-related deaths could be avoided using MCED screens to detect cancers in 50–79 year-olds in the United States [9].

However, whilst there are now a small number of multi-cancer tests with real potential in terms of early detection [10–12], none are yet proven. Furthermore, even if tests such as this do show utility in a clinical setting, there are question marks over placing too much emphasis on them. For example, any multi-cancer test will ideally need to identify the tissue of origin following a positive result to avoid costly additional tests to locate the cancer. There will be no ‘magic bullet’ solution to the early detection problem—and MCED tests would not solve everything. This means CRUK needs to focus on the whole continuum of challenges in early diagnosis—from discovery research, through translation to regulation and implementation in clinical pathways, as articulated in our Roadmap.

## Remaining challenges

Detecting cancer with a non-invasive liquid biopsy approach is an incredibly exciting prospect for cancer diagnostics. But there are many challenges still to overcome.

In early-stage cancers, the low and variable amount of biomarkers raises the simple problem that different blood samples from the same individual might yield different results. Low biomarker amounts also dictate that liquid biopsy techniques must be highly sensitive; however, the high sensitivity needed to detect ctDNA—or other biomarkers—can affect the specificity of the test. There is a concern that benign mutations could well trigger false-positive results [13].

Personalised approaches, such as the INVAR work, which can increase sensitivity, operate by targeted sequencing across a patient-specific list of mutations—and are, as such, not well suited for early detection or diagnosis of new cancers. Currently, the cost of sequencing would also be prohibitive for utilisation as a screening tool. New ways of increasing sensitivity need to be developed for the potential of liquid biopsies in screening and early detection to be reached. There are several promising avenues of research on this front—alternate methods of ctDNA analysis including novel epigenetic assay methods (https://www.ludwigcancerresearch.org/news-releases/taps-and-more/?scientist=chunxiao-song), examining fragmentation patterns [14] or combining ctDNA with other molecular marker types such as circulating proteins [11].

Despite the incredible advances made utilising ctDNA as a biomarker for liquid biopsies, there is still a lack of pathophysiologic information that needs to be addressed for a robust understanding of how and when it is released from tumours. We also need more discovery and translational research to help us understand the biological nature of the early lesions detected by liquid biopsy (which may differ from those detected through conventional means)—what is their prognosis and how should we optimally treat or monitor them?

Another vital issue is the way in which prospective screening and early diagnostic tests are evaluated. Studies using cancer patients who already have a diagnosis are likely to overestimate sensitivity. This is because those patients are likely to have received a symptomatic diagnosis and are therefore likely to have a more advanced cancer compared to cancers in the average-risk population. To address this, we need studies that evaluate the effectiveness of any liquid biopsy-based screen or early detection test to be conducted prospectively using the target population—i.e. those who have yet to experience symptoms. This, of course, raises further questions over who would get these tests, how to make that cost-effective and how best to evaluate MCEDs. The usual criterion of impact on cancer-specific mortality for a new screening test would be practically impossible to reach for a test that aims to detect 50 cancer types. For a screening test to be implemented, there must be a demonstrable cancer-specific mortality benefit of using a given test. For example, fewer people die of lung cancer as a result of introducing a lung cancer test. Based on the very low and varying cancer incidence rates for different cancer types (and their subsequent mortalities) in a screening population, a screening trial for a diagnostic test for one cancer would need to be designed to not only find enough cases of that cancer in the general population but also to allow follow-up on those people over a long enough period of time to see the effect of the test on mortality. And this is just for a single cancer. If you have a multi-cancer detection test and if you were to demonstrate the gold standard cancer-specific mortality endpoint for all of the cancers that the test detects, you would need enough incident cases of each cancer and enough deaths specifically from each cancer to show a significant difference. This would necessitate a trial with a massive number of people, beyond reasonable constraints of cost and feasibility. As such, we may need to consider alternate ways to evaluate these MCEDs.

## Towards the future

Whilst treatments for cancer have made revolutionary steps forward, early detection research has made comparatively limited progress, presenting a persistent scientific and clinical challenge.

Now, with advances in technology starting to open-up new research paths, a test that could reliably pick up signs of cancer before it takes hold is a clear goal of early detection research. Liquid biopsies clearly have huge potential here, and a growing evidence base in the literature shows that the level of interest in the research community is high. Along with other early detection approaches, (and potentially integrated with them) research into liquid biopsies is a key part of our strategy. We welcome more consideration not only into further technological development, but how such tests will be translated and trialled in real-world clinical settings. Indeed, CRUK has made early detection a key priority, and with our roadmap and the significant research funding opportunities we have made available in this space, we hope to stimulate and enable researchers in the field to deliver a future where early diagnosis of cancer is a routine reality.
